# Unit-based Scorecards Result in Improved Bundled Resuscitation for Septic Patients in a Single Freestanding Children’s Hospital

**DOI:** 10.1097/pq9.0000000000000513

**Published:** 2021-12-16

**Authors:** Jeremy M. Loberger, Jessica Hicks, Sherry Green, Brenda Vason

**Affiliations:** From the * Department of Pediatrics, University of Alabama at Birmingham, Birmingham, Ala.; † Children’s of Alabama, Department of Performance Improvement, Birmingham, Ala.

## Abstract

The Children’s Hospital Association’s Improving Pediatric Sepsis Outcomes (IPSO) collaborative is a multi-center quality improvement (QI) learning collaborative of 61 U.S. children’s hospitals that seeks to improve sepsis outcomes through collaborative learning and reliable implementation of evidence-based interventions in pediatric emergency departments, intensive care units, general care units, and hematology/oncology units. Specifically, IPSO’s goals are to decrease sepsis-attributable mortality and prevent hospital-onset sepsis among children.

The following 10 abstracts represent a select group of projects undertaken by IPSO participating hospitals that were presented at one of three collaborative events in 2020 and 2021. IPSO’s Research Workgroup reviewed all submitted abstracts and selected the top 10 for inclusion in this Supplement

## Background:

Bundled resuscitation including timely fluid resuscitation and antibiotic administration improves mortality in children with sepsis. Two of the central challenges of implementing a hospital-wide quality improvement initiative are addressing the unique obstacles in widely varying care environments and delivering timely performance data.

## Objectives:

In April 2019, our sepsis team implemented a unit-based scorecard reporting system in an effort to provide unit directors with personalized and actionable data regarding the care of septic children in their particular unit.

## Methods:

**Fig. 1.**
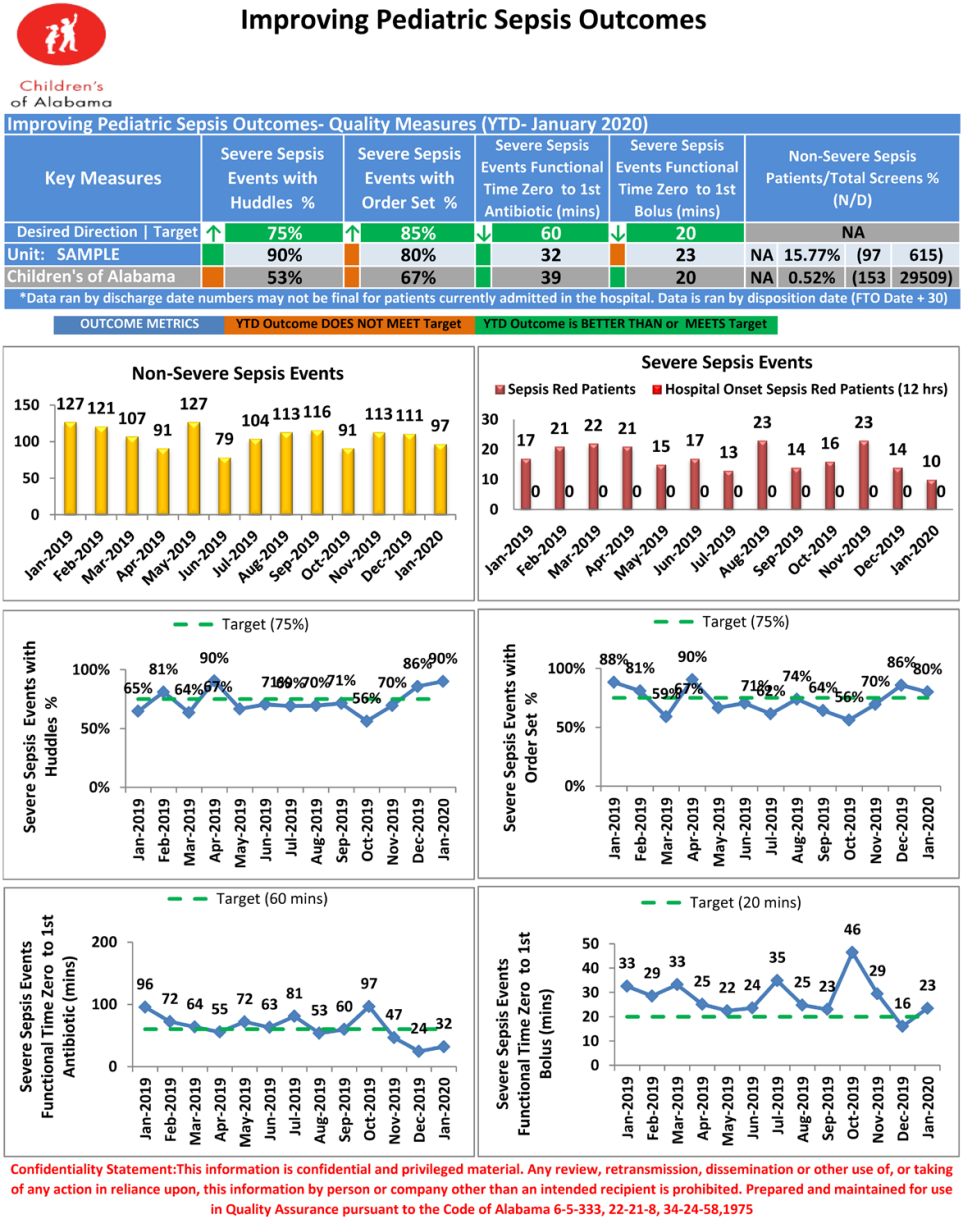
Sample unit-based scorecard.

The scorecards contain hospital-wide as well as unit-specific performance data for four of the five key Improving Pediatric Sepsis Outcomes process measures—huddles, order set utilization, time to first IV antibiotic, and time to first fluid bolus (Fig. 1). Specific patient encounters are also reported to assist apparent cause analysis efforts. No other interventions occurred concurrently.

## Results:

Before implementation, we met or exceeded the less than 60-minute threshold for intravenous antibiotic administration for 1 month and the less than 20-minute threshold for first fluid bolus in zero months. Following implementation, we met or exceeded the antibiotic threshold in six out of 9 months (67%) and the fluid bolus threshold in three out of 9 months (33%). The average time to first antibiotic decreased by 28% (89 to 64 minutes) after implementation. The average time to first fluid bolus decreased by 32% (38 to 26 minutes) after implementation.

## Conclusions:

Providing timely, specific, and actionable information at the unit level can lead to meaningful system wide improvements in care delivery to septic children.

